# Astragaloside IV Promotes Adult Neurogenesis in Hippocampal Dentate Gyrus of Mouse through CXCL1/CXCR2 Signaling

**DOI:** 10.3390/molecules23092178

**Published:** 2018-08-29

**Authors:** Fei Huang, Yunyi Lan, Liyue Qin, Huaihuai Dong, Hailian Shi, Hui Wu, Qinrui Zou, Zhibi Hu, Xiaojun Wu

**Affiliations:** Shanghai Key Laboratory of Compound Chinese Medicines, The Ministry of Education (MOE) Key Laboratory for Standardization of Chinese Medicines, The State Administration of TCM (SATCM) Key Laboratory for New Resources and Quality Evaluation of Chinese Medicine, Institute of Chinese Materia Medica, Shanghai University of Traditional Chinese Medicine, Shanghai 201203, China; Fei_H@hotmail.com (F.H.); ddlanyunyi@126.com (Y.L.); 13774474958@163.com (L.Q.); 13917511681@163.com (H.D.); shihailian2003@163.com (H.S.); zgykdxwuhui@foxmail.com (H.W.); zqr314@hotmail.com (Q.Z.)

**Keywords:** astragaloside IV, neurogenesis, CXCL1/CXCR2, early proliferative cells, proliferative radial gila-like cells, newly generated neurons

## Abstract

Astragaloside IV (ASI) has been reported to promote neural stem cells proliferation in vitro and CXCR2 expression on neutrophils. The present study was aimed to investigate the influence of ASI on adult neurogenesis in hippocampal dentate gyrus (DGs) of mouse and to discuss the possible underlying mechanisms. Total number of proliferative cells (BrdU^+^), pre-mature neurons (DCX^+^), early proliferative cells (BrdU^+^/DCX^+^), proliferative radial gila-like cells (BrdU^+^/GFAP^+^) and newly generated neurons (BrdU^+^/NeuN^+^) after ASI or vehicle administration for two weeks were counted, respectively. The results showed that BrdU^+^ cells and DCX^+^ cells were significantly increased in DGs of mice administered with ASI. The numbers of BrdU^+^/DCX^+^, BrdU^+^/GFAP^+^ cells and BrdU^+^/NeuN^+^ cells were also elevated in the ASI group. Correspondingly, ASI increased the protein expression of hippocampal DCX, GFAP and NeuN. Further study disclosed that ASI remarkably up-regulated the mRNA and protein expressions of CXCL1 as well as that of CXCR2 in the hippocampus. The promotive effect of ASI on DCX, GFAP and NeuN protein expression was abolished by SB225002, the inhibitor of CXCR2. Our results indicated that ASI modulated the homeostasis of the CXCL1/CXCR2 signaling pathway, which might be responsible for the increased neurogenesis within the hippocampal DGs of mice.

## 1. Introduction

Adult hippocampal neurogenesis attracts specific attention as it is suggested to play an important role in higher cognitive function, most notably memory processes, and certain affective behaviors [1]. Lateral ventricles and hippocampal dentate gyrus (DG) are the well-known regions in the brain where the neural progenitor cells proliferate and differentiate into mature neurons throughout the lifetime of animals [2,3]. In the hippocampus, the mature neurons can consolidate surrounding structures and participate in many critical processes such as learning and memory [4]. Accumulative evidence shows that adult neurogenesis is essential for specific types of hippocampus-dependent learning [5,6,7].

Chemokines (chemotactic cytokines) compose a family of small protein ligands involved in leukocyte migration and communication [8]. Interestingly, chemokines and their receptors are expressed in all major types of cells in the central nervous system (CNS), and a growing body of evidence suggests that chemokines and their receptors also mediate the cellular communication in CNS [9]. For instance, CXCR2 has been shown to enhance the survival of hippocampal neurons [10,11], and is involved in patterning the spinal cord by controlling the position of oligodendrocyte precursors after stimulation by its ligand CXCL1 [12]. 

Astragaloside IV (ASI) is one of the major active saponins in *Astragalus membranaceus* (Fisch) Bge, a widely used herb in China for the treatment of cardiovascular, hepatic, and renal disorders [13]. Several studies demonstrated that ASI has a prominent antioxidant effect shown by inhibition of the generation of reactive oxygen species (ROS) [14], reduction of lipid peroxidation [15], and elevation of antioxidant enzymes [16]. It exerts neuroprotective effects against ischemic brain injury by anti-oxidation [17], anti-inflammation [18], anti-apoptosis [19] and blood–brain barrier protection [20]. In addition, our studies demonstrated that it can attenuate experimental autoimmune encephalomyelitis in mice by counteracting oxidative stress at multiple levels [21]. Recently, it was reported that ASI ameliorates the learning and memory deficit in rats after chronic cerebral hypoperfusion [22] and attenuates cognitive impairments induced by transient cerebral ischemia and reperfusion in mice [23]. Moreover, ASI promotes neural stem cells proliferation and differentiation [24]. An in vitro study showed that application of ASI apparently promotes CXCR2 expression on LPS-induced neutrophils [25]. However, whether ASI can benefit neurogenesis in vivo has not been demonstrated yet. 

In the present study, the effect of ASI on neurogenesis in the DGs of mice was investigated by bromodeoxyuridine (BrdU) assay. Serial brain sections from mice were then double stained with antibodies against BrdU^+^ doublecortin (DCX, a pre-mature neuron marker), BrdU^+^ glial fibrillary acidic protein (GFAP, an astroglial marker) or BrdU^+^ neuronal nuclei (NeuN, a neuronal marker). Meanwhile, hippocampal expressions of genes related with neurogenesis were analyzed with the PCR array method and confirmed by real-time PCR. The results showed that ASI actively participated in hippocampal neurogenesis, which was closely associated with enhanced CXCL1/CXCR2 signaling transduction. The study deepened our understanding of the role of ASI in the dynamic and complex process of neurogenesis that is connected to the normal brain function.

## 2. Results

### 2.1. ASI Increased the Total Number of Proliferative Cells (BrdU^+^), Pre-Mature Neurons (DCX^+^) and Early Proliferative Cells (BrdU^+^/DCX^+^)

**Figure 1:**
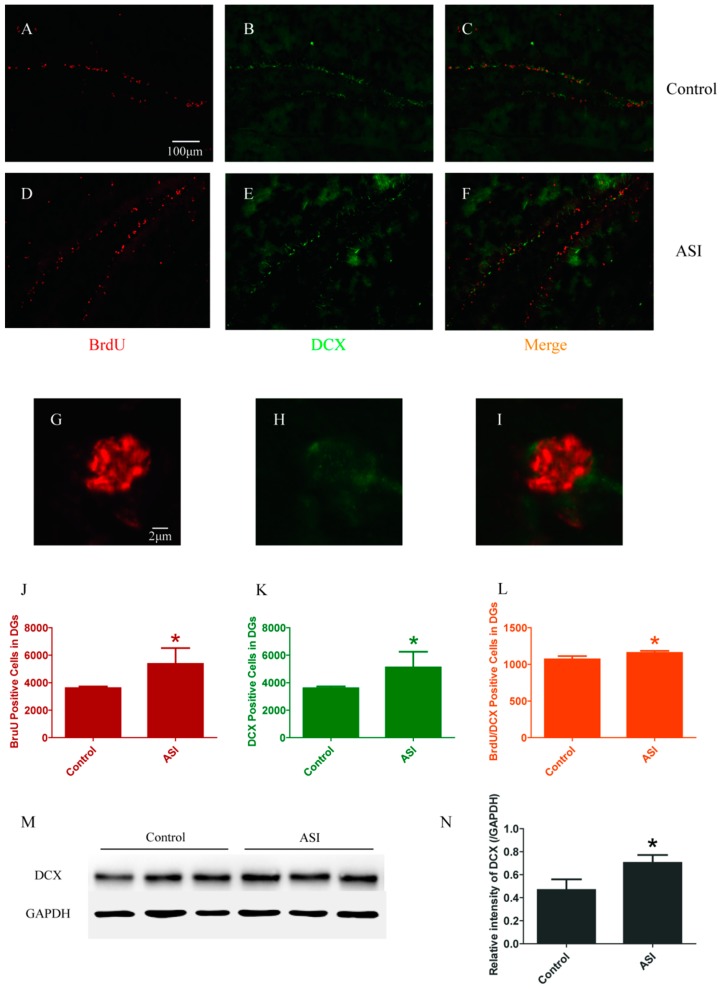
ASI (25 mg/kg) increased the total number of proliferative cells (BrdU^+^), pre-mature neurons (DCX^+^) and early proliferative cells (BrdU^+^/DCX^+^). (**A**–**I**) Confocal images of BrdU (red) and DCX (green) immunostaining: (**A**–**C**) control group; (**D**–**F**) ASI group; and (**G**–**I**) enlarged images of the cells from ASI group. (**A**–**F**) Scale bar = 100 μm. (**G**–**I**) Scale bar = 2 μm. (**J**–**L**) Quantification of BrdU^+^, DCX^+^ and BrdU^+^/DCX^+^ immunopositive cells in two groups (*n* = 4/group). (**M**) Western blotting analysis of DCX in hippocampus. (**N**) Gray intensity analysis of DCX in hippocampus (*n* = 8/group). * *p* < 0.05. The data are presented as mean ± SD.

For neurogenesis analysis, BrdU-immunopositive cells and DCX-immunopositive cells were counted firstly. The DGs of ASI group mice contained more BrdU-immunopositive cells (Control vs. ASI, respectively, *n* = 4 per group, *p* < 0.05) (Figure 1A,D,G,J) and DCX-immunopositive cells (Control vs. ASI, respectively, *n* = 4 per group, *p* < 0.05) (Figure 1B,E,H,K) compared to that of their controls. Moreover, double immunopositive (BrdU^+^/DCX^+^) cells showed a difference between two groups (Control vs. ASI, respectively, *n* = 4 per group, *p* < 0.05) (Figure 1C,F,I,L). Moreover, the total amount of DCX protein was elevated in the hippocampus of ASI group mice (*n* = 8 per group) (Figure 1M,N). These results indicated that ASI increased proliferative cells, pre-mature neurons and early proliferative cells.

### 2.2. ASI Increased the Total Number of Proliferative Radial Glia-Like Cells (BrdU^+^/GFAP^+^) and Newly Generated Neurons (BrdU^+^/NeuN^+^) 

**Figure 2:**
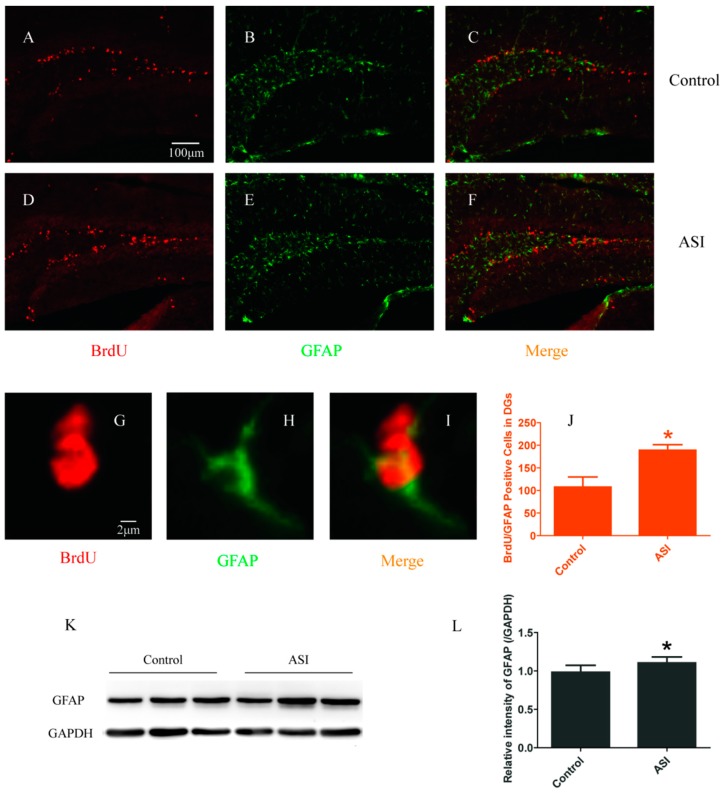
ASI (25 mg/kg) increased the total number of proliferative radial glia-like cells (BrdU^+^/GFAP^+^). (**A**–**I**) Confocal images of BrdU (red) and GFAP (green) immunostaining: (**A**–**C**) Control group; (**D**–**F**) ASI group; and (**G**–**I**) enlarged images of the cells from ASI group. (**A**–**F**) Scale bar = 100 μm. (**G**–**I**) Scale bar = 2 μm. (**J**) Quantification of BrdU^+^/GFAP^+^ double immunopositive cells in two groups (*n* = 4/group). (**K**) Western blotting analysis of GFAP in hippocampus. (**L**) Gray intensity analysis of GFAP in hippocampus (*n* = 8/group). * *p* < 0.05. The data are presented as mean ± SD.

**Figure 3:**
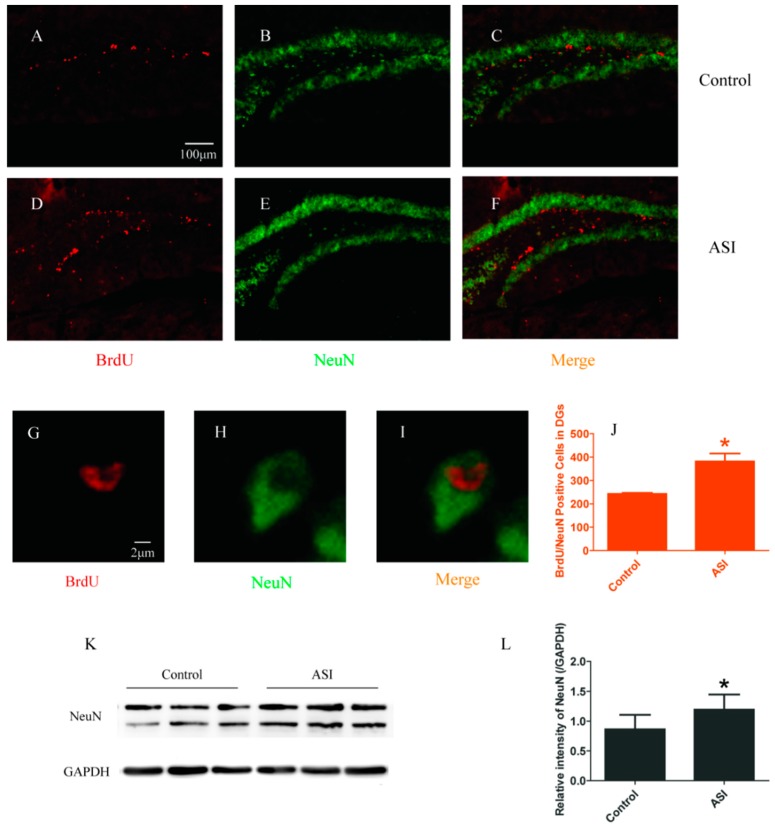
ASI (25 mg/kg) increased the total number of newly generated neurons (BrdU^+^/NeuN^+^). (**A**–**I**) Confocal images of BrdU (red) and NeuN (green) immunostaining: (**A**–**C**) Control group; (**D**–**F**) ASI group; and (**G**–**I**) enlarged images of the cells from ASI group. (**A**–**F**) Scale bar = 100 μm. (**G**–**I**) Scale bar = 2 μm. (**J**) Quantification of BrdU^+^/NeuN^+^ double immunopositive cells in two groups (*n* = 4/group). (**K**) Western blotting analysis of NeuN in hippocampus. (**L**) Gray intensity analysis of NeuN in hippocampus (*n* = 8/group). * *p* < 0.05. The data are presented as mean ± SD.

To examine the effect of ASI on proliferative radial glia-like cells and newly generated neurons in DGs, BrdU^+^/GFAP^+^ and BrdU^+^/NeuN^+^ immunopositive cells were counted, respectively. Confocal microscopic results showed that GFAP^+^ and NeuN^+^ cells were abundant in the granular cell layer of the DGs in both groups (Figure 2B,E,H and Figure 3B,E,H). Meanwhile, the numbers of BrdU^+^ cells with GFAP (Control vs. ASI group, respectively, *n* = 4 per group, *p* < 0.05) (Figure 2C,F,I,J) and NeuN (Control vs. ASI group, respectively, *n* = 4 per group, *p* < 0.05) (Figure 3C,F,I,J) expression were significantly increased in the ASI group mice. Consistently, the total amount of GFAP and NeuN proteins were also elevated remarkably in the hippocampus in ASI group mice (*n* = 8 per group, *p* < 0.05) (Figure 2K,L and Figure 3K,L). These results indicated that ASI increased proliferative gila-like cells and newly generated neurons in the subgranular zone (SGZ). 

**Figure 4:**
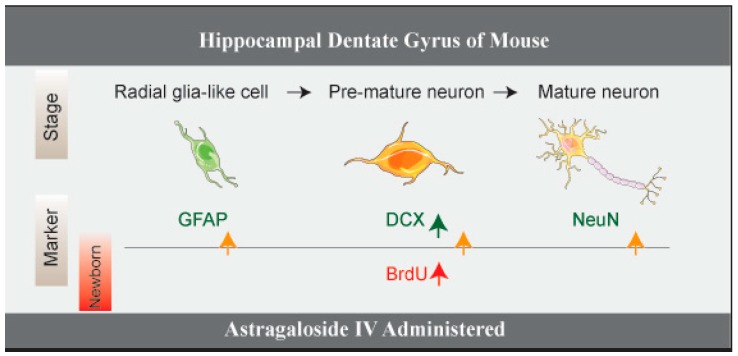
The schematic illustration of the promotive effect of ASI (25 mg/kg) on adult neurogenesis in DGs.

In summary, all above results suggested that ASI increased adult neurogenesis at multiple levels in DGs (Figure 4).

### 2.3. ASI Enhanced CXCL1/CXCR2 Signaling Pathway

**Figure 5:**
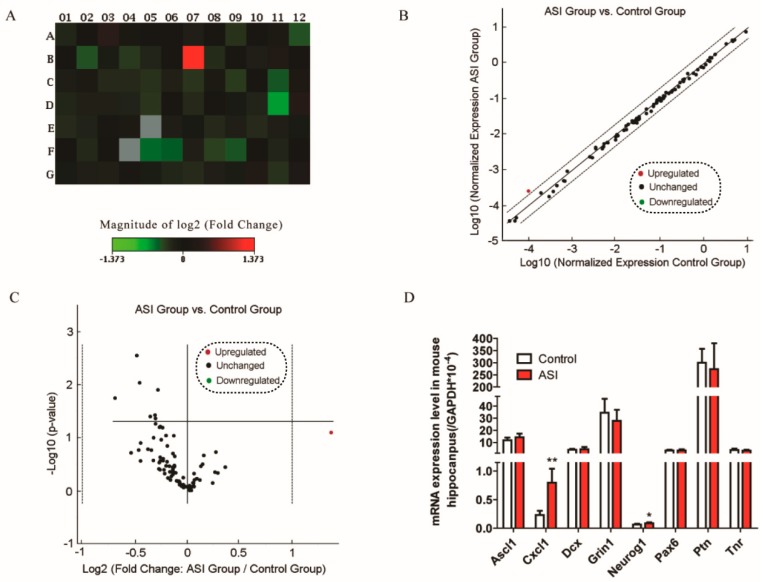
The effect of ASI (25 mg/kg) on eighty-four neurogenesis related genes. (**A**) The heat map provides a graphical representation of fold change between two groups overlaid onto PCRarray plate layout; (**B**) The scatter plot compares the normalized expression of every gene on the array between two groups by plotting one against another to quickly visualize gene expression changes. The central line indicated unchanged gene expression; (**C**) The volcano plot displays statistical significance versus fold-change on the *y*- and *x*-axes, respectively, enabling identification of genes with significant changes; (**D**) Verification of mRNA expression levels in mouse hippocampus; *n* = 6/group. * *p* < 0.05; ** *p* < 0.01. The data are presented as mean ± SD.

**Figure 6:**
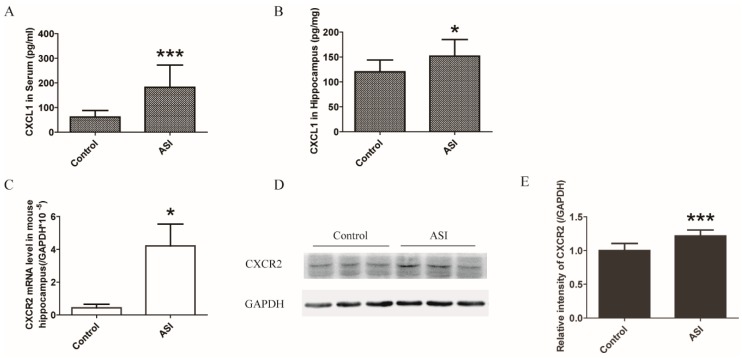
ASI (25 mg/kg) promoted neurogenesis through CXCL1/CXCR2 signaling pathway. (**A**,**B**) ASI promoted protein expression of CXCL1 in mouse serum and hippocampus (*n* = 10/group); (**C**) ASI enhanced CXCR2 mRNA expression level in mouse hippocampus (*n* = 6/group); (**D**) Western blotting analysis of CXCR2 in hippocampus; (**E**) Gray intensity analysis of CXCR2 in the hippocampus of mice (*n* = 8/group). * *p* < 0.05; *** *p* < 0.001. The data are presented as mean ± SD.

The pathways involved in neurogenesis in mouse hippocampus by ASI were investigated in a pilot study including 84 neurogenesis-related genes (Figure 5 and Table 1). The preliminary data showed that mRNA of eight genes were differently expressed after ASI treatment judged by *p*-value (Figure 5A–C). However, further quantitative PCR did not perfectly corroborate the significant fold-change of these genes (Figure 5D) as only CXCL1 displayed a 3.4-fold elevation. In addition, ASI promoted the protein expression of CXCL1 in mouse serum and hippocampus (Figure 6A,B). Since CXCL1 signals through its receptor CXCR2, the mRNA and protein expressions of the receptor were examined. Not surprisingly, hippocampal CXCR2 mRNA and protein expressions were markedly up-regulated by ASI compared to the control (Figure 6C–E). These results suggested the modulation of ASI on CXCL1/CXCR2 signaling pathway. 

### 2.4. ASI Promoted Neurogenesis through CXCL1/CXCR2 Signaling Pathway

**Figure 7:**
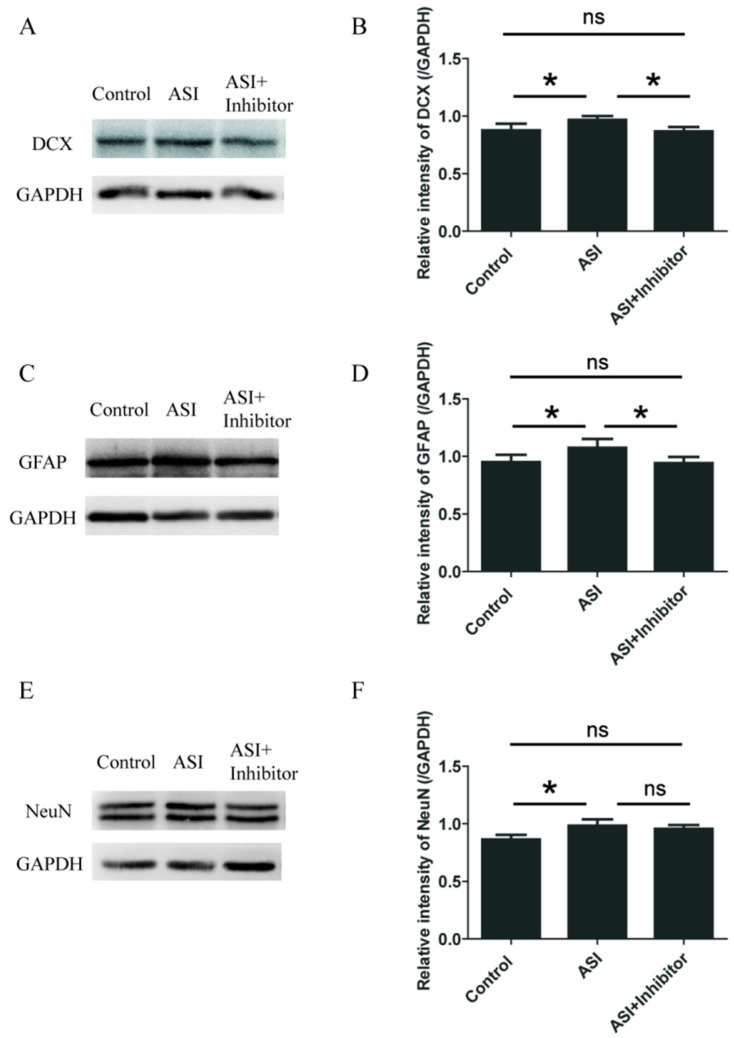
CXCR2 inhibitor abrogated the effects of ASI (25 mg/kg) on proliferative cells of adult neurogenesis and newly generated neurons. (**A**,**C**,**E**) CXCR2 inhibitor abolished the effect of ASI on DCX, GFAP and NeuN in the hippocampus of mice. (**B**,**D**,**F**) Gray intensity analysis of DCX, GFAP and NeuN in the hippocampus of mice (*n* = 5/group). * *p* < 0.05. The data are presented as mean ± SD.

To determine whether the effect of ASI was mediated through CXCL1/CXCR2, we utilized SB225002, an inhibitor of CXCR2. Similar to previous results, ASI treatment resulted in the significant increase of hippocampal DCX, GFAP and NeuN protein expression (Figure 7A–F). However, SB225002 reversed the promotive effects of ASI on DCX and GFAP (Figure 7A–D), and partly reversed the promotive effects of ASI on NeuN (Figure 7E,F), suggesting that ASI enhanced neurogenesis through CXCL1/CXCR2 signaling pathway.

## 3. Discussion

In the present study, the effects of ASI on the hippocampal neurogenesis of mice were examined. Our data showed that ASI increased adult neurogenesis in the DGs, which was mediated by regulating the signaling transduction through the CXCL1/CXCR2 system. 

Adult hippocampal neurogenesis is a complex process, in which new excitatory granule cells are generated in the DGs, particularly in the subgranular zone (SGZ). Hippocampal neurogenesis originates from a population of neuronal precursor cells in the SGZ [26]. They give rise to intermediate progenitor cells with glial or neuronal phenotype [1]. In our experiments, BrdU^+^ cells and DCX^+^ cells were significantly increased in the ASI group of mice. Meanwhile, the numbers of BrdU^+^/DCX^+^ cells, BrdU^+^/GFAP^+^ cells and BrdU^+^/NeuN^+^ cells were also increased by ASI treatment, suggesting a promotive effect of the compound on adult neurogenesis at multiple levels (Figure 4). Although the radial glia-like cells (also called type 1 hippocampal progenitors) express the astrocyte marker GFAP, these cells are morphologically and functionally different from mature astrocytes. The expression of GFAP protein in the hippocampus could not distinguish between them.

Several lines of evidence indicate that CXCL1/CXCR2 signaling plays an important role in neurogenesis. For instance, CXCL1 stimulation enhances the proliferative response of the rat’s immature spinal cord oligodendrocyte precursors to platelet derived growth factor [27,28] as well as elevates the number of dopaminergic neurons in rat ventral midbrain precursor and neurosphere cultures [29]. Normally functioning CXCR2 seems to protect neurons from injury. In brain trauma, neuronal CXCR2 downregulation is suggested to render neurons more vulnerable to injury [30]. While CXCR2 ligands macrophage inflammatory protein 2 (MIP-2), CXCL1 and CXCL8 are indicated to protect hippocampal neurons against beta-amyloid (1–42) induced death [31]. It has been reported that CXCR2 antagonist SB225002 significantly attenuated microglial activation and blood brain barrier (BBB) damage, increased myelination, and reduced astrogliosis in the white matter [32]. As a result, we considered that SB225002 could pass through the BBB. Our experiments showed that ASI boosted hippocampal CXCL1 and CXCR2 expression at both mRNA and protein levels. In the presence of CXCR2 inhibitor, the effect of ASI on the protein expression of DCX, GFAP and NeuN could be abrogated more or less. Therefore, the enhanced CXCL1/CXCR2 signaling transduction at least partly led to the promotive effects of ASI on adult neurogenesis in the hippocampal DGs of mice. 

One limitation of the present study is that we did not evaluate the characteristics of all types of newborn cells to conclusively ascertain the early markers for neuronal lineage. Moreover, it remains to be determined about how the actions of ASI regulated the adult neurogenesis through CXCL1/CXCR2 signaling transduction. There was another neurogenesis related gene Neurog 1 showed a significant change in expression between the treated and control (Figure 5D) groups, but due to its minimal change, we would like invest it in the future.

Our results indicated that ASI modulated the homeostasis of the CXCL1/CXCR2 system, which might be responsible for the increased neurogenesis in the hippocampal DGs of mice. Neurogenesis of the adult brain is a very interesting phenomenon. Many studies over the last 20 years have aimed to decipher the role of the phenomenon in the brain [33]. With so many functions ascribed to the adult brain neurogenesis, ASI may have a beneficial effect on the recovery of brain dysfunction.

## 4. Materials and Methods

### 4.1. Animal and Drug Administration

Male C57BL/6 mice (18–22 g, 6 weeks old) were provided by the Laboratory Animal Center of Shanghai University of Traditional Chinese Medicine (SHUTCM, Shanghai, China). The mice were housed under a 12 h light/12 h dark cycle at room temperature (25 ± 1 °C) and fed with food and water *ad libitum*. All experiments on animals were performed according to the protocol approved by Animal Care and Use Committee of SHUTCM and all animals received humane care (Ethical approval no. SZY201607003). Half of the mice were injected intraperitoneally (i.p.) with ASI (25 mg/kg, 40% 1,2-Propanediol + 1% Polyethylene glycol + 5% Ethanol in phosphate buffer saline solution, i.p.) for two weeks, while the other half of mice served as the control and were administered with solvent. Among the animals, eight mice (four in control group and four in ASI group) used for IHC analysis were additionally treated with 5-bromo-2′-deoxyuridine (BrdU, Sigma Aldrich, St. Lpuis, MO, USA, cat#B5002,) to label endogenous proliferating cells. The BrdU treated mice were injected intraperitoneally with BrdU (50 mg/kg) once every two days for two weeks, and were sacrificed 18 h following the last BrdU injection. To evaluate the influence of CXCR2 inhibitor on the promotive effect of ASI, a potent and selective antagonist of CXCR2 (SB225002, Selleck, Houston, TX, USA) was used (4 mg/kg) together with ASI for two weeks. At last, the mice after drug treatment were sacrificed and their hippocampi were dissected, snap frozen in liquid nitrogen and stored at −80 °C until analysis. 

### 4.2. Immunohistochemistry

For the histological analysis, animals were anesthetized with 2% pentobarbital sodium and perfused transcardially with 0.1 M phosphate buffer (PBS, pH 7.4) followed by 4% paraformaldehyde. The brains were dissected and fixed in the same fixative overnight. Brain tissues were dehydrated by infiltration with 10% and 30% sucrose, respectively, for 24 h at 4 °C. Then, they were serially sectioned into 20 µm coronal slices containing the dentate gyrus of hippocampus from Bregma −1.00 to −2.92 mm according to the mouse brain atlas [34]. For each mouse, there were ninety-six slices, sixteen of which, with an interval of 120 µm, were chosen for immunohistochemistry analysis. The final total positive cell numbers in each mouse were calculated as six-fold the sum of that in the sixteen slices.

To examine the cell proliferation and differentiation, double immunofluorescence staining for BrdU and DCX/GFAP/NeuN was performed. The free-floating sections were washed with PBS, immersed in 10% donkey serum and 0.3% Triton X-100 in 0.1 M PBS for 1 h, and incubated with rabbit anti-DCX antibody (1:400; *v/v*, Santa Cruz Biotechnology, Santa Cruz, CA, USA, cat#4604)/mouse anti-GFAP antibody (1:400; Santa Cruz Biotechnology, cat#3670)/rabbit anti-NeuN antibody (1:400; Santa Cruz Biotechnology, cat#12943) at 4 °C overnight. The sections were then incubated with Alexa-labeled donkey anti-rabbit or anti-mouse antibody at room temperature for 1 h. For BrdU immunostaining, the sections were incubated in 2N HCl for 30 min at 37 °C to denature DNA, and neutralized in 0.1 M borate buffer (pH 8.4) for 10 min. After being washed with PBS, the sections were incubated with 0.1% trypsin for 5 min at 37 °C followed by 10% donkey serum and 0.3% Triton X-100 in 0.1 M PBS. Thereafter, the sections were incubated with rat anti-BrdU antibody (1:150; Bio-Rad, Hercules, CA, USA, cat#OBT0030G) at 4 °C overnight, then washed with PBS and incubated with Alexa-labeled donkey anti-rat antibody at room temperature for 1 h. Finally, all sections were washed and mounted on slides using gold anti-fade reagent with DAPI (Life Technologies, Gaithersburg, MD, USA, cat#P36935). The fluorescent pictures were taken with the confocal microscope system (FV10i Fluo view, Olympus, Japan).

### 4.3. RT^2^ Profiler PCR Array Analysis

Mouse Neurogenesis RT^2^ Profiler PCR arrays were carried out on 384-well plates containing primers for 84 pathway/disease/function genes related with neurogenesis, 5 house keeping genes, 1 genomic DNA contamination control, 3 reverse transcription quality controls and 3 PCR reaction quality controls. Total RNA was extracted from hippocampus using RNasy^®^ Mini Kit (Qiagen, Duesseldorf, Germany, cat#74104) and reverse transcribed into cDNA using RT^2^ First Strand kit (Qiagen, cat#330401). cDNA was mixed with RT^2^ SYBR Green/ROX PCR MasterMix (Qiagen, cat#330521). The mixture was subsequently added into each well of the 384-well plates (Qiagen, cat#PAMM-404Z) and quantitative PCR was performed. Data was analyzed using 2^^(−CT)^ method.

### 4.4. Real-Time PCR

To confirm the immunohistochemistry and PCR arrays’ results, total RNAs from the hippocampi of mice were extracted using Trizol according to the manufacturer’s instructions (Life Technologies, Gaithersburg, MD, USA). The RNAs were then reversely transcribed into cDNA with Revert Aid First Strand cDNA Synthesis kit (Fermentas, Burlington, ON, Canada). The synthesized cDNA was used as templates for quantitative real-time PCR with Universal SYBR Green/ROX qPCR Master Mix (Roche, Basel, Switzerland). Primers used are listed in Table 2. 

### 4.5. Western Blotting Analysis

To examine the effect of ASI on the expression of proteins, samples from hippocampi were homogenized, sonicated, and subjected to Western blotting analysis. Twenty micrograms of proteins from each sample were separated on 10–15% SDS-PAGE. After being transferred onto PVDF membranes, the proteins were incubated with respective primary antibodies against DCX (1:1000, *v/v*), GFAP (1:1000, *v/v*), NeuN (1:1000, *v/v*), CXCR2 (1:1000; *v/v*, Abcam cat#14935) horseradish peroxidase-conjugated secondary antibodies sequentially as described previously [21]. The protein bands were visualized by an ECL-prime kit and quantified with ImageJ 1.46r software (NIH, Bethesda, MD, USA).

### 4.6. ELISA Analysis

To detect the effect of ASI on CXCL1, concentrations of mouse chemokine in serum and hippocampi were measured using a CXCL1-specific ELISA kit (Boster Biological Technology, Wuhan, China), following the manufacturer’s instructions. 

### 4.7. Statistical Analysis

All data are presented as mean ± SD. The difference of measurement data was evaluated by unpaired t-test and one-way ANOVA with Tukey multiple comparison test; the difference of count data was evaluated by Kruskal–Wallis test. SPSS 18.0 (SPSS Inc., Chicago, IL, USA) was used for analysis. The value of *p* < 0.05 was regarded as statistically significant. 

## 5. Conclusions

We have demonstrated that that ASI promoted adult neurogenesis in hippocampal dentate gyrus of mouse, which was mediated by regulating the signaling transduction through the CXCL1/CXCR2 system. Further studies of the effect of ASI on neurogenesis-related brain function or behavior are needed in order to replicate our observations and to extend the evidence on the effect of therapy on CXCL1/CXCR2 system. Therefore, ASI may be a potential therapeutic drug for the recovery of brain dysfunction.

## Figures and Tables

**Table 1:** The effect of ASI on mRNA expressions of eighty-four neurogenesis related genes.

Description	Symbol	Fold Change	*p*-Value
Acetylcholinesterase	Ache	0.857	0.408505
Adenosine A1 receptor	Adora1	1.016	0.958737
Adenosine A2a receptor	Adora2a	1.2858	0.364259
Anaplastic lymphoma kinase	Alk	0.9632	0.831172
Amyloid beta (A4) precursor protein-binding, family B, member 1	Apbb1	1.0156	0.930706
Apolipoprotein E	Apoe	1.0062	0.874886
Amyloid beta (A4) precursor protein	App	0.9095	0.20132
Artemin	Artn	1.0458	0.845847
Achaete-scute complex homolog 1 (Drosophila)	Ascl1	0.808	0.038118
B-cell leukemia/lymphoma 2	Bcl2	1.0486	0.632784
Brain derived neurotrophic factor	Bdnf	0.9419	0.678017
Bone morphogenetic protein 2	Bmp2	0.734	0.277842
Bone morphogenetic protein 4	Bmp4	1.0426	0.725701
Bone morphogenetic protein 8b	Bmp8b	0.7345	0.127788
Cyclin-dependent kinase 5, regulatory subunit 1 (p35)	Cdk5r1	0.9182	0.33772
CDK5 regulatory subunit associated protein 2	Cdk5rap2	0.7886	0.173978
Cholinergic receptor, muscarinic 2, cardiac	Chrm2	0.8612	0.481537
CAMP responsive element binding protein 1	Creb1	1.0151	0.998111
Chemokine (C-X-C motif) ligand 1	Cxcl1	2.5899	0.081082
Doublecortin	Dcx	0.8235	0.012748
Discs, large homolog 4 (Drosophila)	Dlg4	0.9898	0.847781
Delta-like 1 (Drosophila)	Dll1	0.9084	0.501594
Dopamine receptor D2	Drd2	1.1828	0.723812
Dishevelled 3, dsh homolog (Drosophila)	Dvl3	1.0458	0.480882
Ephrin B1	Efnb1	0.9166	0.35916
Epidermal growth factor	Egf	0.9504	0.69603
E1A binding protein p300	Ep300	0.8368	0.062456
V-erb-b2 erythroblastic leukemia viral oncogene homolog 2, neuro/glioblastoma derived oncogene homolog (avian)	Erbb2	0.8359	0.236367
Fibroblast growth factor 2	Fgf2	0.8105	0.055528
Filamin, alpha	Flna	0.8888	0.349493
Glial cell line derived neurotrophic factor	Gdnf	0.7991	0.271871
Glucose phosphate isomerase 1	Gpi1	0.946	0.668135
Glutamate receptor, ionotropic, NMDA1 (zeta 1)	Grin1	0.7829	0.040383
Histone deacetylase 4	Hdac4	1.0155	0.813732
Hairy and enhancer of split 1 (Drosophila)	Hes1	0.725	0.173203
Hairy/enhancer-of-split related with YRPW motif 1	Hey1	1.1153	0.21879
Hairy/enhancer-of-split related with YRPW motif 2	Hey2	0.8526	0.251846
Hairy/enhancer-of-split related with YRPW motif-like	Heyl	0.8945	0.635531
Interleukin 3	Il3	0.88	0.582914
Midkine	Mdk	0.8764	0.145008
Myocyte enhancer factor 2C	Mef2c	0.8003	0.101506
Myeloid/lymphoid or mixed-lineage leukemia 1	Kmt2a	1.0275	0.997062
Microtubule-associated protein 2	Map2	0.8362	0.276201
Necdin	Ndn	1.0382	0.718334
Norrie disease (pseudoglioma) (human)	Ndp	0.9318	0.657985
Neurogenic differentiation 1	Neurod1	0.8694	0.344025
Neurogenin 1	Neurog1	0.62	0.018182
Neurogenin 2	Neurog2	1.2238	0.468483
Neurofibromatosis 1	Nf1	0.8321	0.06433
Noggin	Nog	0.875	0.288031
Notch gene homolog 1 (Drosophila)	Notch1	1.0654	0.599656
Notch gene homolog 2 (Drosophila)	Notch2	0.9508	0.77945
Nuclear receptor subfamily 2, group E, member 3	Nr2e3	1.0185	0.936243
Neuron-glia-CAM-related cell adhesion molecule	Nrcam	0.9158	0.460346
Neuregulin 1	Nrg1	0.9564	0.765114
Neuropilin 1	Nrp1	0.835	0.406853
Neuropilin 2	Nrp2	0.9089	0.406554
Neurotrophin 3	Ntf3	0.831	0.299471
Netrin 1	Ntn1	0.9076	0.44445
Odd Oz/ten-m homolog 1 (Drosophila)	Tenm1	0.9365	0.665725
Oligodendrocyte transcription factor 2	Olig2	1.0327	0.853541
Platelet-activating factor acetylhydrolase, isoform 1b, subunit 1	Pafah1b1	0.8419	0.110867
Par-3 (partitioning defective 3) homolog (C. elegans)	Pard3	0.976	0.878636
Paired box gene 3	Pax3	1.0185	0.936243
Paired box gene 5	Pax5	0.6895	0.195764
Paired box gene 6	Pax6	0.7163	0.002868
POU domain, class 3, transcription factor 3	Pou3f3	0.8996	0.542927
POU domain, class 4, transcription factor 1	Pou4f1	0.7709	0.167186
Pleiotrophin	Ptn	0.7296	0.009374
RAS-related C3 botulinum substrate 1	Rac1	0.9743	0.778785
Roundabout homolog 1 (Drosophila)	Robo1	0.8688	0.092542
Reticulon 4	Rtn4	1.0437	0.826015
S100 calcium binding protein A6 (calcyclin)	S100a6	0.9265	0.466286
S100 protein, beta polypeptide, neural	S100b	0.9084	0.453236
Sonic hedgehog	Shh	0.8914	0.445715
Slit homolog 2 (Drosophila)	Slit2	1.0868	0.636415
Superoxide dismutase 1, soluble	Sod1	0.9206	0.228334
SRY-box containing gene 2	Sox2	0.8523	0.274519
SRY-box containing gene 3	Sox3	0.8288	0.392865
Signal transducer and activator of transcription 3	Stat3	0.9089	0.366775
Transforming growth factor, beta 1	Tgfb1	0.9192	0.351884
Tyrosine hydroxylase	Th	1.2009	0.452233
Tenascin R	Tnr	0.8102	0.04399
Vascular endothelial growth factor A	Vegfa	1.107	0.320206

The red number means *p* < 0.05.

**Table 2:** Sequences of primers for quantitative PCR.

Gene	Forward Primer	Reverse Primer
Ascl1	GTCACAAGTCAGCGGCCAAGCA	TTCTTGTTGGCCGCGCCGTT
CXCL1	GCCAATGAGCTGCGCTGTCAGT	AAGGCAAGCCTCGCGACCATTC
CXCR2	ATGCTGTCCCATGCCACTCAGAGA	CCATTTACTTTAGATGCAGCCCAGACA
Dcx	CATCTAGAAATATGAGAGGGTCACGGATG	TCTTCCAGTTCATCCATGCTTCCAAT
Neurog1	CCTCTCCGGGGCATCGAATGTT	TGAGCTTGGTGTCGTCGGGGAA
GAPDH	ATGTGTCCGTCGTGGATCTGA	ATGCCTGCTTCACCACCTTCT
Grin1	CAAGCCCAACGCCATACAGATGG	AGCAACGTCTCCAGGCGCTTCT
Pax6	CCAGGGCAATCGGAGGGAGTAA	CGCCCATCTGTTGCTTTTCGCTA
Ptn	GCAACGTAGAAAATTTGCAGCTGCCTTC	TCTCTGAGTCTTCATGGTCTGTTTGCAC
Tnr	AGGTGACTACAGAAAGGGCTCAGAGACA	GCTCAGCAGTTCCTGCAGTACCTGG
